# Supplementary material for: Pre hospital delay and its associated factors in acute myocardial infarction in a developing country
Source: PLoS One. 2021 Nov 24;16(11):e0259979. doi: 10.1371/journal.pone.0259979 (PMC8612565; doi:10.1371/journal.pone.0259979)
Supplement: Attachment — Submitted filename: Response to Reviewers2.docx [file pone.0259979.s002.docx]

**Thanks to the reviewer for such a wonderful feedback. Once again, not only has these comments have helped this manuscript to be technically more sound, it also taught me a lot. We have attempted to revise as per the comments. I hope and pray that these revisions would suffice for the article to be eligible for publication.**

**Response to Reviewers:**

**Comment :** 1) P values need to be removed from OR estimate sin the abstract and the results/discussion. You can leave them in the regression table, but it is most appropriate to interpret OR along with 95% CI's.
** You will notice for example that one of your variables was significant at the 0.03 level but the interval included 1, meaning although statistically significant it could not be as influential as others which did not include 1 in the interval.

**Response:** Thanks for this comment, another lesson learnt. The p-values have been removed accordingly from the text and only the ORs with their 95% CI have been mentioned.

**Comment:** 2) Did your logistic regression include two outcomes? If not, then the regression that was conducted was a multivariable logistic regression, not a multivariate. Univariate logistic regression is a fine model selection to builds a final model, but realize that including all variables in a model may change the associations observed in univariate models. SOI in this way a forward or backward variable selection process maybe more appropriate than using univariate logistic regression to identify influential variables.
** The wording for multivariate needs changed. The other comment doesn't need included in the manuscript, other than to point out in the discussion that there are potential confounders not included in bivariate models.

**Response:** I am sorry but as a junior researcher I am not sure whether I understood this comment correctly. Our logistic regression analysis included two outcomes so we kept the term “multivariate”. The wording for the multivariate regression analysis have been changed a little bit.

3) Although the authors include a paragraph which mentions previous studies, they do not present specific findings from these in the introduction. This change is needed to enhance the readers experience and understanding for the current state of myocardial infarction in that country.

**Response:** I apologize for not mentioning this. Findings have been added for reference 4 accordingly in introduction.
